# The role of 4DCT in the localization of parathyroid adenomas in primary hyperparathyroidism: a retrospective cohort study

**DOI:** 10.1186/s13244-025-02133-z

**Published:** 2025-11-06

**Authors:** Scott Klerkx, Brigitte Decallonne, Jeroen Meulemans, Lennert Boeckxstaens, Lesley Cockmartin, Janne Vignero, Robert Hermans

**Affiliations:** 1 Department of Radiology, University Hospitals Leuven, Leuven, Belgium; 2 Department of Imaging and Pathology, KU Leuven-University of Leuven, Leuven, Belgium; 3 Department of Endocrinology, University Hospitals Leuven, Leuven, Belgium; 4 Clinical and Experimental Endocrinology, Department of Chronic Diseases and Metabolism, KU Leuven, Leuven, Belgium; 5 Department of Otorhinolaryngology-Head and Neck Surgery, University Hospitals Leuven, Leuven, Belgium; 6 Department of Oncology, Section Head and Neck Oncology, KU Leuven, Leuven, Belgium; 7 Department of Nuclear Medicine, UZ Leuven, Leuven, Belgium; 8 Department of Imaging and Pathology, Nuclear Medicine and Molecular Imaging, KU Leuven, Leuven, Belgium

**Keywords:** Hyperparathyroidism (parathyroid neoplasms), Four-dimensional computed tomography, Single-photon emission-computed tomography, Ultrasonography, Parathyroidectomy

## Abstract

**Objectives:**

To evaluate the diagnostic value of four-dimensional computed tomography (4DCT) in conjunction with ultrasonography (US) and sestamibi scintigraphy (SeS) with ^99m^Tc-MIBI, compared to US and SeS alone in primary hyperparathyroidism.

**Materials and methods:**

A retrospective study was conducted on patients who underwent 4DCT for suspected parathyroid adenomas between January 2019 and December 2023, followed by surgical exploration. The diagnostic performance of 4DCT, US, and SeS was compared in terms of sensitivity, specificity, positive predictive value (PPV), negative predictive value (NPV), and accuracy. Additionally, enhancement patterns, false-negative and false-positive 4DCT results, and adenoma location in cases with negative US and SeS findings were analyzed.

**Results:**

One hundred forty-six 4DCT studies were included. Overall, adding 4DCT to US and/or SeS demonstrated significantly higher sensitivity (76.4%) and accuracy (75.7%) compared to US (31.8% and 35.7%) and SeS (26.2% and 31.6%) alone. Specificity was highest for SeS (85.7%), followed by 4DCT (78.3%) and US (72.5%). PPV was high for all modalities, with 4DCT achieving 96.1%, while NPV was low (22.4% for 4DCT, 9.8% for US, 10.3% for SeS). False-negative 4DCT results were mostly due to adenoma adherence to the thyroid. 4DCT identified 48 adenomas missed by US and SeS, with 21 located in ectopic sites.

**Conclusion:**

Adding 4DCT to US and/or SeS provides superior sensitivity and accuracy in detecting parathyroid adenomas compared to US and SeS alone. However, negative results should be interpreted cautiously in patients with clinical and biochemical suspicion for primary hyperparathyroidism.

**Critical relevance statement:**

Adding 4DCT to US and SeS offers superior sensitivity and accuracy in detecting parathyroid adenomas, potentially enhancing clinical decision-making—particularly in challenging cases where prior imaging was negative—though negative results should be interpreted with caution.

**Key Points:**

- Adding 4D-CT to US and sestamibi scintigraphy (SeS) significantly improves the detection of parathyroid adenomas over US and SeS alone.
- 4D-CT provides the highest agreement with surgical findings, improving surgical planning.
- 4D-CT effectively identifies adenomas in ectopic locations, often missed by US and SeS.
- The most common cause of false-negative 4D-CT results is adenoma adherence to the thyroid, misread as thyroid tissue.
- Negative imaging results across all modalities warrant cautious interpretation.

**Graphical Abstract:**

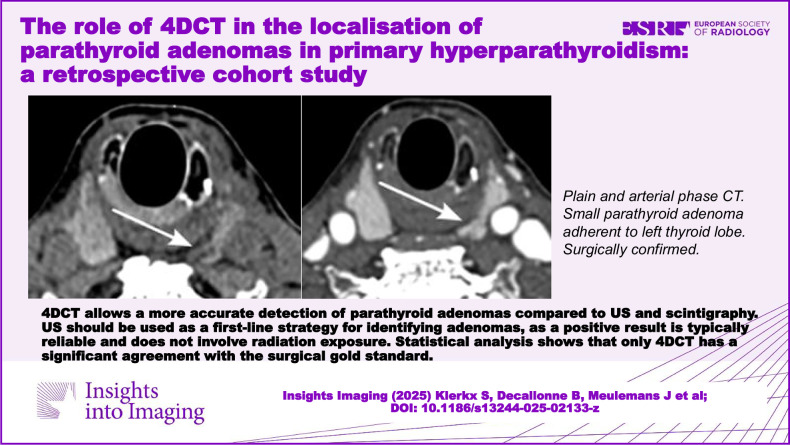

## Introduction

Parathyroid adenomas are benign tumors of the parathyroid glands, which regulate calcium levels through the secretion of parathyroid hormone (PTH). They are the leading cause of primary hyperparathyroidism (PHPT), a condition marked by elevated blood calcium levels due to autonomous PTH secretion. PHPT can cause fatigue, muscle weakness, kidney stones, and gastrointestinal issues, and may lead to complications such as fractures due to osteoporosis and renal insufficiency if left untreated. Most cases (75–85%) are caused by a single adenoma; multigland disease accounts for 10–15% of cases. Parathyroid carcinoma is very rare (~1%) [1, 2].

Surgical removal is the primary treatment for parathyroid adenomas. While bilateral neck exploration (BNE) was once the standard approach, improved preoperative localization methods have enabled the use of minimally invasive parathyroidectomy (MIP). MIP offers benefits such as smaller incisions, reduced operative time, decreased overall hospital stay, and improved cosmetic outcomes, without compromising surgical success [3].

Preoperative localization techniques are critical for successful MIP. Common methods include ultrasonography (US) and sestamibi scintigraphy (SeS) with ^99m^Tc-MIBI. On US, the adenoma typically appears as a homogeneous hypoechoic nodule, often with a characteristic enlarged feeding artery [4]. SeS detects hyperactive parathyroid glands based on enhanced ^99m^Tc-MIBI uptake in these structures, with single-photon emission computed tomography (SPECT) and SPECT/CT providing additional anatomical information [5].

Four-dimensional computed tomography (4DCT), introduced in 2006, combines anatomical imaging with functional contrast changes over time, improving the ability to distinguish parathyroid adenomas from other neck structures [6]. Despite promising results, no consensus has been reached regarding the optimal imaging strategy for the preoperative localization of parathyroid adenomas. To further evaluate the role of 4DCT, we conducted a retrospective study of all patients who underwent 4DCT for PHPT at our institution between January 2019 and December 2023 and subsequently underwent surgical exploration.

## Materials and methods

### Patients

Data were retrospectively collected upon institutional review board approval for all patients with primary hyperparathyroidism who underwent 4DCT between January 1, 2019, and December 31, 2023, for diagnosing and localizing parathyroid adenomas, and who had surgery prior to January 1, 2025, with histopathological assessment if tissue was removed. Both 4DCT and surgery were performed at University Hospitals Leuven, Belgium, a tertiary referral center.

A total of 214 4DCT examinations were collected. Of these, 68 examinations were excluded for the following reasons: (1) no surgery and histopathology prior to January 1, 2025 (*n* = 54), (2) incorrect scanning protocol (*n* = 11), and (3) study performed for tumor follow-up after parathyroid carcinoma resection (*n* = 3). As a result, 146 4DCT studies were included. The majority of these patients also underwent US (*n* = 142) and SeS (*n* = 115), either at our institution or elsewhere. Of the 142 US studies, 97 (68%) were performed at our institution, and of the 115 SeS studies, 75 (65%) were performed at our institution.

### Imaging protocols and assessment

#### Imaging protocols

The 4DCT scanning protocol consisted of a non-contrast topogram and an initial unenhanced scan, after which intravenous contrast injection was administered (75 mL at 4 mL/s, followed by a saline chaser). Post-contrast imaging was acquired at 30 s (arterial phase) and 90 s (delayed phase) after the start of the injection. The scan range extended from the lower teeth to the carina, ensuring full coverage of the cervical and upper thoracic regions. In all patients, native slice thickness was 1 mm; multiplanar reformatting was used in all patients.

The nuclear imaging protocol in our institution involved a dual-isotope scintigraphy with ^99m^Tc-MIBI and ^123^I. Dual-isotope imaging was performed at least 2 h after administering 10 MBq ^123^I and 5 min after injecting 550 MBq ^99m^Tc-MIBI. Planar imaging of the neck and mediastinum was conducted first, followed by a dual-isotope SPECT/CT scan with a low-dose CT component. Digital subtraction of the ^123^I and ^99m^Tc-MIBI images enabled visualization of hyperfunctioning parathyroid tissue.

For the US examinations at our institution, a linear transducer (2–9 MHz) was used for superficial structures, and a microconvex transducer (4–20 MHz) was employed for superior depth penetration.

#### Imaging assessment

All 4DCT examinations were performed at our institution and reviewed by both a senior radiology resident in training and an experienced head and neck radiologist. US examinations at our institution were performed by a senior radiology resident under the supervision of an experienced head and neck radiologist, and SeS studies at our institution were reviewed by an experienced specialized nuclear medicine physician, with or without prior review by a senior nuclear medicine resident. Most externally performed US and SeS scans were conducted at secondary care centers by a general radiologist and a general nuclear medicine physician, respectively.

### Surgical procedure

Depending on the results of preoperative imaging, patients underwent MIP or bilateral neck exploration. The location of the glands was recorded by the quadrant of the neck in which they were found: parathyroid A (left superior), B (left inferior), C (right inferior), D (right superior), or an ectopic location. During surgery, intra-operative PTH (IOPTH) monitoring was performed to verify the adequate removal of a hyperfunctional parathyroid. The resected parathyroid adenomas were sent to the pathology department for frozen section analysis to obtain intra-operative histological confirmation. Specimens underwent subsequent definitive pathological examination.

### Data and statistical analysis

#### Outcome measures of 4DCT compared to US and SeS

The radiological reports were compared with the operative and histopathological findings as the gold standard, after which the true-positive, true-negative, false-positive, and false-negative rates were calculated on a per-lesion basis. For patients who also underwent US or SeS, the same outcome metrics were calculated for these modalities. This was performed first for the total patient group, second for all patients with single-gland and multigland disease, and third in patients with a prior history of thyroid or parathyroid surgery. Additionally, the results of 4DCT in patients with negative results from US, SeS, or both modalities combined were evaluated as well.

Statistical analysis was performed to assess the diagnostic performance of the three imaging modalities compared to histopathology. The primary outcome measures—sensitivity, specificity, positive predictive value (PPV), negative predictive value (NPV), and accuracy—together with the corresponding 95% confidence intervals (CIs) were calculated for each imaging modality and for the different patient groups separately. Accuracy was defined as the proportion of correctly classified cases (true positives and true negatives) among all evaluated cases.

#### Enhancement patterns in 4DCT

The enhancement pattern of all confirmed parathyroid adenomas was determined as described by Bahl et al in 2015 [7]. All patterns required the adenoma to exhibit lower attenuation than the thyroid gland on non-enhanced images. With type A, the lesion was higher in attenuation than the thyroid gland in the arterial phase and might vary in the delayed phase. With type B, the lesion was not higher in attenuation than the thyroid gland in the arterial phase and was lower in attenuation than the thyroid gland in the delayed phase. Type C lesions showed attenuation that was neither higher than the thyroid gland in the arterial phase nor lower than the thyroid gland in the delayed phase. Lesions not fitting in one of these categories were labeled as ‘unclassifiable.’

All lesions were classified according to these relative enhancement patterns by two readers, a senior radiology resident and an experienced head and neck radiologist. The readers assigned the patterns subjectively and did not measure attenuation values. If discrepancies were observed in the documented attenuation patterns, the studies were reviewed, and consensus was reached.

#### Retrospective analysis of false-negative 4DCT reports

All false-negative 4DCT scans were retrospectively reviewed by the same two readers. For the adenomas that could retrospectively be identified, the enhancement patterns were determined, and the reasons for initially missing the adenomas were analyzed.

#### Tissue origin of false-positive 4DCT cases

In cases where resected tissue was incorrectly identified as hyperfunctional parathyroid tissue on 4DCT, the histopathological characteristics were assessed.

#### Location of adenomas not visualized on US and SeS

The locations of all confirmed parathyroid adenomas that were not detected on either US or SeS were analyzed to assess whether they were more frequently found in atypical ectopic sites.

#### Total effective dose of 4DCT compared to SeS

The effective dose for the 4DCT studies was estimated using the CT dose calculation software CT-Expo (v2.7) [8]. For each combination of CT scanner model and tube voltage, a conversion factor (k-factor) was determined based on a reference male phantom. Conversion factors were normalized using the scan length and volume computed tomography dose index values specific to each scanner and protocol setting. For each patient, the effective dose (in mSv) was calculated by multiplying the patient-specific dose-length product (DLP) by the corresponding scanner- and voltage-specific conversion factor (0.0114–0.0149 mSv/mGy.cm).

The total effective dose for the SeS studies was calculated as the sum of the contributions from both radiopharmaceuticals (^99m^Tc-MIBI and ^123^I) and the low-dose CT component. For each tracer, the effective dose was calculated by multiplying the administered activity by its specific dose conversion factor (0.12 mSv/MBq for ^123^I and 0.015 mSv/MBq for ^99m^Tc-MIBI), and the total ED of the radiopharmaceuticals was obtained by summing both results. For the low-dose CT component, the effective dose was calculated by multiplying the patient-specific DLP by the weighted average value of the scanner- and voltage-specific conversion factors (0.0133 mSv/mGy.cm).

For patients with complete datasets from both the 4DCT and SeS examinations required for dose calculation, mean values and mean absolute deviation (MAD) were calculated, and a paired *t*-test was performed to assess dose differences between the 4DCT and SeS examinations.

## Results

### Surgical and histopathological findings

**Fig. 1:**
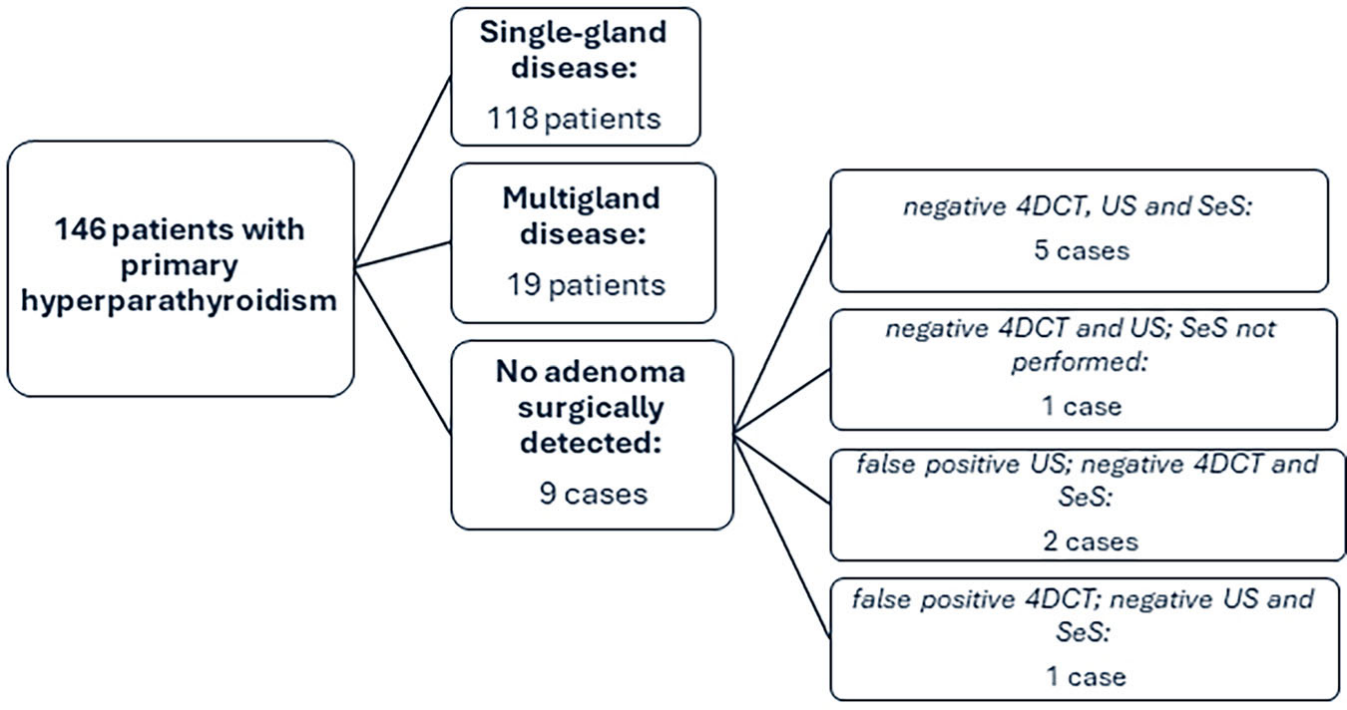
Flowchart of included patients with surgical results

A total of 146 patients who all underwent a 4DCT scan were included, in whom 161 hyperfunctional parathyroid lesions were surgically and histopathologically confirmed. Among these cases, single-gland disease was identified in 118 cases and multigland disease in 19 cases. No adenoma could be surgically confirmed in 9 cases: in 6 of these cases, all imaging studies performed were negative; in 2 cases, US was falsely positive (with negative 4DCT and SeS); and in 1 case, 4DCT yielded a false-positive result (with negative US and SeS) (Fig. 1).

In 19 cases (24 adenomas), previous surgery had been performed on the thyroid or parathyroid gland. In 3 cases, a parathyroid carcinoma was detected on histopathological examination.

### Outcomes of 4DCT compared to US and SeS

All comparisons of 4DCT, US, and SeS were performed on a per-lesion basis. As shown in Table 1, in the total patient group, 4DCT demonstrated the highest sensitivity of 76.4%, compared to US (31.8%) and SeS (26.2%). Specificity was highest for SeS at 85.7%, compared to 75.0% for US and 68.8% for 4DCT. PPV was high across all modalities, with 4DCT achieving 96.1%, US 92.8%, and SeS 94.9%. NPV was low for all modalities, with 4DCT showing the highest value (22.4%), compared to US (9.8%) and SeS (10.3%). Overall accuracy was highest for 4DCT at 75.7%, compared to US (35.7%) and SeS (31.6%).

**Table 1:** Outcomes of 4DCT compared to US and SeS

	Sensitivity % [95% CI]	Specificity % [95% CI]	PPV % [95% CI]	NPV % [95% CI]	Accuracy % [95% CI]
Total patient group (*n* = 161)					
4DCT	76.4% [69.07–82.72]	68.8% [41.34–88.98]	96.1% [92.24–98.09]	22.4% [15.76–30.73]	75.7% [68.71–81.83]
US	31.8% [24.55–39.80]	75.0% [47.62–92.73]	92.8% [84.23–96.88]	9.8% [7.44–12.84]	35.7% [28.52–43.40]
SeS	26.2% [18.68–34.96]	85.7% [57.19–98.22]	94.9% [83.26–98.58]	10.3% [8.30–12.73]	31.6% [23.89–40.10]
Single-gland disease (*n* = 118)					
4DCT	73.7% [64.83–81.40]	N/A	100% [95.85–100.00]	N/A	N/A
US	34.8% [26.14–44.23]	N/A	100% [91.19–100.00]	N/A	N/A
SeS	24.7% [16.37–34.76]	N/A	100% [85.18–100.00]	N/A	N/A
Multigland disease (*n* = 43)					
4DCT	83.7% [69.30–93.19]	N/A	100% [90.26–100.00]	N/A	N/A
US	23.1% [11.13–39.33]	N/A	100% [66.37–100.00]	N/A	N/A
SeS	31.0% [15.28–50.83]	N/A	100% [66.37–100.00]	N/A	N/A
Prior surgery (*n* = 24)					
4DCT	83.3% [62.62–95.26]	N/A	100% [83.16–100.00]	N/A	N/A
US	31.8% [13.86–54.87]	N/A	100% [59.04–100.00]	N/A	N/A
SeS	36.4% [17.20–59.34]	N/A	100% [63.06–100.00]	N/A	N/A

*4DCT* four-dimensional computed tomography, *US* ultrasonography, *SeS* sestamibi scintigraphy, *PPV* positive predictive value, *NPV* negative predictive value, *CI* confidence interval

4DCT also showed the highest sensitivity in the subgroups of patients with single-gland disease (73.7%), multigland disease (83.7%), and in patients with prior thyroid or parathyroid surgery (83.3%). Since there were no true negatives or false positives in these subgroups, specificity, NPV, and accuracy could not be calculated.

### Outcomes of 4DCT in cases with prior negative imaging

As in the previous section, all comparisons were performed on a per-lesion basis. In patients with a negative US (*n* = 106), 4DCT demonstrated a sensitivity of 68.8%, specificity of 64.3%, and an accuracy of 69.2%, as shown in Table 2. In cases with negative SeS (*n* = 90), sensitivity was 67.8%, specificity 76.9%, and accuracy 69.0%. When both US and SeS were negative (*n* = 65), 4DCT showed a sensitivity of 61.5%, specificity of 72.7%, and accuracy of 63.1%. Across all groups, PPV was consistently high (≥ 93%), but NPV was low, ranging from 22.5 to 26.3%.

**Table 2:** Outcomes of 4DCT in cases with prior negative imaging

	Sensitivity % [95% CI]	Specificity % [95% CI]	PPV % [95% CI]	NPV % [95% CI]	Accuracy % [95% CI]
4DCT in cases with negative US (*n* = 106)	68.8% [60.13–78.35]	64.3% [35.14–87.24]	93.5% [87.53–96.70]	22.5% [15.15–32.07]	69.2% [60.07–77.26]
4DCT in cases with negative SeS (*n* = 90)	67.8% [57.10–77.25]	76.9% [46.19–94.96]	95.2% [87.82–98.17]	26.3% [18.95–35.24]	69.0% [59.09–77.72]
4DCT in cases with negative US and SeS (*n* = 65)	61.5% [48.64–73.35]	72.7% [39.03–93.98]	93.3% [83.82–97.37]	23.5% [16.07–33.11]	63.1% [51.26–73.89]

*4DCT* Four-dimensional computed tomography, *US* ultrasonography, *SeS* sestamibi scintigraphy, *PPV* positive predictive value, *NPV* negative predictive value, *CI* confidence interval

### Enhancement pattern

The enhancement patterns of the 139 proven parathyroid adenomas on 4DCT were predominantly Type B (94/139; 68%), followed by Type A (23/139; 17%) and Type C (13/139; 9%), as shown in Table 3. Nine adenomas (6%) were not classifiable, either due to previous total thyroidectomy (6/9), abnormal or pathological thyroid parenchyma (2/9), or because they did not appear hypodense compared to the thyroid parenchyma on non-enhanced images (1/9).

**Table 3:** Enhancement patterns of pathology-proven parathyroid adenomas on 4DCT

Enhancement pattern	*N* = adenomas (*N* = 139)	False-negatives (retrospectively visualized) (*N* = 16)	False positives (*N* = 5)
Type A	23/139 (17%)	0/16	2/5
Type B	94/139 (68%)	11/16 (69 %)	3/5
Type C	13/139 (9%)	3/16 (19 %)	0
Not classifiable	9/139 (6%)	2/16 (13 %)	0
Previous total thyroidectomy	6/9		
Abnormal/pathological thyroid parenchyma	2/9		
Adenoma not hypodense compared to the thyroid parenchyma on the non-enhanced images	1/9		

*4DCT* four-dimensional computed tomography

Among the 16 initially missed but retrospectively visualized adenomas, most lesions also showed a Type B enhancement pattern (11/16; 69%), followed by Type C (3/16; 19%). None exhibited a type A pattern, and two of the missed adenomas (13%) were not classifiable.

Of the five false-positive lesions where other tissue was misinterpreted as a parathyroid adenoma, three exhibited a Type B enhancement pattern and two showed a Type A pattern, while none were Type C or unclassifiable.

### Causes of false-negative 4DCT results

**Fig. 2:**
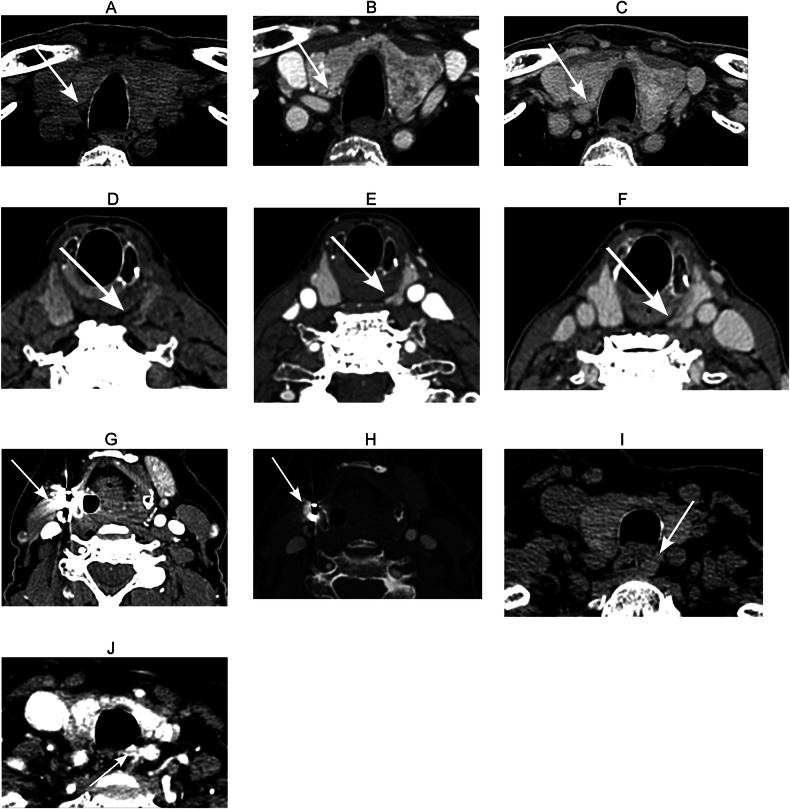
Examples of initially missed parathyroid adenomas on 4DCT in four patients. **A**–**C** Non-contrast (**A**), arterial phase (**B**) and delayed phase (**C**) images showing a parathyroid adenoma closely adherent to the right thyroid lobe, initially misinterpreted as thyroid tissue. The lesion appears slightly hypodense relative to the thyroid parenchyma on non-contrast CT and isoattenuating relative to the thyroid on the arterial phase and delayed phase (type C enhancement pattern). **D**–**F** Non-contrast (**D**) arterial phase (**E**) and delayed phase (**F**) images showing another parathyroid adenoma adherent to the left thyroid lobe, also initially misinterpreted as thyroid tissue; the lesion is slightly hypodense on non-contrast CT and isoattenuating on the arterial phase and delayed phase relative to the thyroid (type C enhancement pattern). **G** Arterial phase image of a parathyroid adenoma located anterior to the common carotid artery, not visualized due to metal artifact following prior thyroidectomy. **H** Same image with adjusted window settings, enabling retrospective visualization of the parathyroid adenoma. **I**, **J** Non-contrast (**I**) and arterial phase (**J**) images of a paraesophageal parathyroid adenoma, not detected because of motion artifact on the arterial phase

Out of the 38 false-negative 4DCT cases, 16 adenomas (42%) were retrospectively detected by correlating with the surgical findings (Table 4, Fig. 2). The mostcommon reason was strong adherence of the adenoma to the thyroid parenchyma, leading to misinterpretation as thyroid tissue (*n* = 9). Additional single cases were missed due to motion artifact, material artifact, and an atypical enhancement pattern (not hypodense on non-contrast images). Furthermore, one adenoma was missed because it was adherent to another parathyroid adenoma, while another adenoma was misinterpreted as normal parathyroid tissue. Moreover, two cases involved ectopically located parathyroid adenomas that were missed.

**Table 4:** Causes of false-negative 4DCT with retrospective visualization

	No. of adenomas
• Strongly adherent to thyroid parenchyma—interpreted as thyroid tissue	9
• Motion artifact	1
• Material artifact	1
• Atypical enhancement pattern (not hypodense on NCCT)	1
• Adherent to another parathyroid adenoma	1
• Misinterpreted as normal parathyroid	1
• Missed ectopically located adenoma	2

*4DCT* four-dimensional computed tomography, *NCCT* non-contrast computed tomography

The remaining 22 adenomas (58%) could not be visualized even in retrospect and were considered definitive false negatives on 4DCT.

### Tissue origin of false-positive lesions

**Fig. 3:**
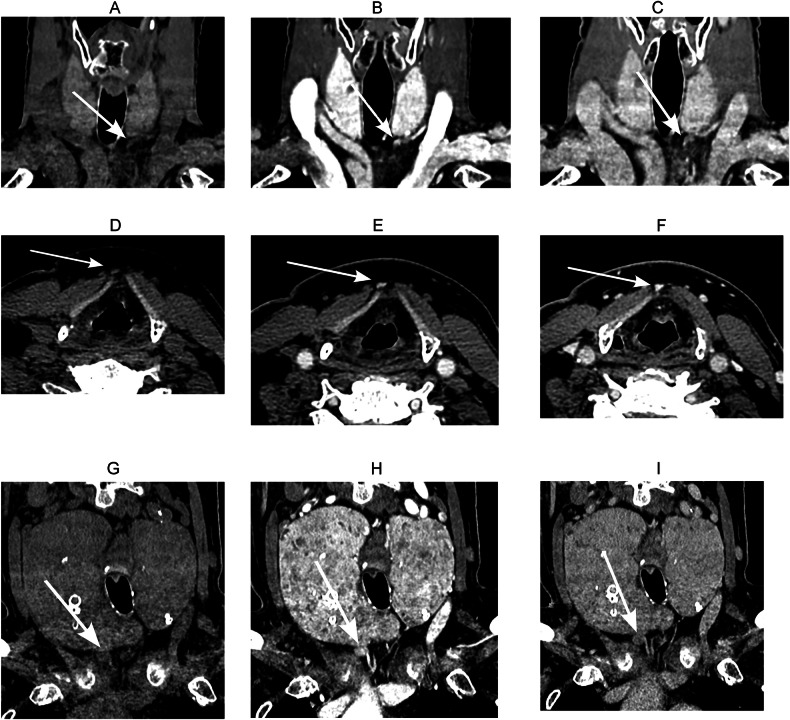
Examples of false-positive 4DCT studies in parathyroid adenoma localization in three patients. **A**–**C** Coronal non-contrast (**A**), arterial phase (**B**) and delayed phase (**C**) images showing a lesion caudal to the left thyroid lobe with type B enhancement pattern, histopathologically confirmed as normal parathyroid tissue with co-resected thymic tissue. **D**–**F** Axial non-contrast (**D**), arterial phase (**E**) and delayed phase (**F**) images showing an elongated, oval-shaped lesion anteromedial to the right thyroid cartilage with type A enhancement pattern (thyroid not shown), histopathologically identified as ectopic thyroid tissue. **G**–**I** Coronal non-contrast (**G**), arterial phase (**H**) and delayed phase (**I**) images showing a lesion caudal to the right thyroid lobe with type C enhancement pattern; histopathology was consistent with a lymph node exhibiting marked sinus histiocytosis

In our dataset, five false-positive lesions were identified on 4DCT. Two of these were histologically classified as normal parathyroid tissue, two as thyroid tissue, and one was found to be a lymph node (Fig. 3).

### Location of adenomas not visualized on US and SeS

A total of 48 adenomas were not detected on either US or SeS but could be visualized on 4DCT. 27 adenomas were found adherent to the thyroid, with 16 located posteriorly, 10 caudally, and 1 anteriorly to the thyroid. Additionally, 21 adenomas were located in ectopic regions, including 10 in the paraesophageal area, 3 in the paratracheal region, and 4 in the tracheoesophageal groove. Single cases were found adjacent to the brachiocephalic trunk (1), common carotid artery (1), in an ectopic anterocaudal position relative to the thyroid (1), and in the hemithyroidectomy space.

### Total effective dose of 4DCT compared to SeS

Complete datasets for dose calculation from both the 4DCT and SeS examinations were available for 59 patients. The mean effective dose was 17.97 mSv for 4DCT and 11.68 mSv for SeS (10.48 mSv from both radiopharmaceuticals and 1.19 mSv from the low-dose CT component). The MAD between the two modalities was 6.59 mSv. A paired *t*-test showed that this difference was statistically significant (*p* < 0.0001).

## Discussion

In this retrospective cohort study comparing 4DCT with US and SeS in patients with primary hyperparathyroidism, 4DCT demonstrated significantly higher sensitivity than US and SeS in the overall patient group, as well as in subgroups of patients with single-gland disease, multigland disease, and those who underwent prior neck surgery. The sensitivity of 4DCT remained relatively high in cases with prior negative imaging by US and/or SeS. Out of the 48 adenomas detected on 4DCT but not detected on both US and SeS, 21 adenomas were located ectopically, and 27 adenomas were found adherent to the thyroid. These findings highlight the added value of 4DCT in identifying parathyroid adenomas in atypical anatomical locations or previously treated areas often missed by US and SeS. The identification of adenomas in these challenging locations improves surgical outcomes by enabling more precise localization for resection. As such, 4DCT should be considered an essential adjunct in patients with negative or inconclusive results from conventional imaging, particularly in cases with overt primary hyperparathyroidism and/or the presence of end-organ complications due to chronic disease.

While 4DCT demonstrated the highest sensitivity, its specificity was slightly, but not significantly, lower compared to SeS and US. The high PPV indicates that when 4DCT identifies a lesion, it is highly likely to be true-positive. The consistently high PPV across all patient subgroups further supports the reliability of positive findings on 4DCT.

The NPV was low for all imaging modalities. This emphasizes that negative results should be interpreted cautiously, particularly in patients with overt primary hyperparathyroidism and/or complications. Out of 16 retrospectively identified adenomas on initially false-negative 4DCT studies, the most common cause for false-negativity was strong adherence of adenomas to the thyroid, leading to misinterpretation as thyroid tissue. Less common causes included motion artifacts, atypical enhancement patterns, and adenomas being missed due to their unusual locations or attachment to other adenomas.

The majority of histopathologically confirmed parathyroid adenomas visualized on 4DCT exhibited a type B enhancement pattern (68%). This is consistent with prior research of Bahl et al, suggesting that the type B pattern is the most common enhancement pattern [7].

Lesions with a type A or B pattern could be diagnosed with only two contrast-enhanced phases. However, the lesions with type C pattern would be more difficult to detect and could be missed without the non-enhanced phase.

Furthermore, the false-positive lesions, which were misinterpreted as parathyroid adenomas, exhibited Type B (3/5) or Type A (2/5) patterns, highlighting the overlap between the enhancement characteristics of parathyroid adenomas and other tissues. This underscores the importance of considering additional diagnostic factors, such as anatomical location and clinical history, to avoid misinterpretation.

4DCT demonstrated a higher effective dose compared to SeS, with a mean absolute deviation (MAD) of 6.59 mSv, approximately twice the average annual background radiation exposure in Europe (3.2 mSv) [9]. At these dose levels, there is no risk of deterministic effects. Although stochastic risks such as radiation-induced cancer cannot be entirely excluded, the probability of occurrence remains very low and is considered acceptable in the context of diagnostic imaging [10]. As 4DCT offers substantial diagnostic advantages, the additional exposure may be justified. However, it warrants careful consideration, particularly in younger patients or those requiring repeated imaging, where cumulative radiation dose is a concern. Moreover, 4DCT involves the use of iodinated contrast agents, which may pose additional risks in patients with impaired renal function. Ultrasound (US) is the safest modality, as it does not involve ionizing radiation nor nephrotoxic contrast agents.

Several other factors influence the imaging strategy for diagnosing parathyroid adenomas, including cost and expertise/availability. Although advanced imaging modalities such as 4DCT and SeS with SPECT/CT are associated with higher initial costs, several studies have indicated that their use may ultimately result in cost savings by decreasing the reliance on BNE [11, 12]. Expertise and availability also play a role. Since its introduction in 2006, the use of 4DCT and the expertise in interpreting it have increased [13].

In addition to US, SeS, and 4DCT, several other imaging modalities have been explored for localizing parathyroid adenomas, including ^18^F-fluorocholine PET/CT and 4D–dynamic contrast-enhanced MRI [14, 15]. Future research should focus on comparing the diagnostic performance of these emerging modalities with established techniques, evaluating their added value in complex cases, and determining their cost-effectiveness and impact on clinical outcomes.

This study has several limitations. First, the retrospective design introduces a selection bias, as only patients who underwent both 4DCT and subsequent surgery were included. This excludes patients who were evaluated with only US and/or SeS. Patients with positive findings on US or SeS may proceed directly to surgery without undergoing 4DCT, leading to an underestimation of the diagnostic performance of US and SeS in this study. However, this also likely underestimates the diagnostic value of 4DCT, since patients with easily localizable adenomas often did not undergo 4DCT. As a result, the study population may overrepresent more complex or clinically challenging cases, limiting the generalizability of the findings to a broader population of patients with primary hyperparathyroidism. On the other hand, this study also excludes those who were not referred for surgery, with very mild hyperparathyroidism, likely caused by tiny parathyroid adenomas difficult to detect by imaging.

Additionally, 4DCT is often used as a second- or third-line imaging modality, which may have influenced diagnostic performance. In many cases, the interpreting radiologist had access to the results of prior US and/or SeS, which could have introduced interpretative bias and affected lesion detection rates. Conversely, the higher proportion of complex cases referred for 4DCT, particularly those with non-localization on US and/or SeS or patients who underwent prior thyroid/parathyroid surgery, may have inflated the proportion of difficult-to-diagnose lesions in the study cohort.

The inclusion of US and SeS studies performed at external institutions may have introduced inconsistencies due to differences in imaging protocols, radiotracer administration, equipment quality, and the experience of interpreting radiologists/nuclear medicine specialists. These variations could have influenced lesion detection and diagnostic performance, further limiting the generalizability of the study findings.

Finally, the relatively small number of true-negative and false-positive cases in the overall study group and their absence in the subgroups of single-gland disease, multigland disease and patients with prior neck surgery, limits the ability to fully evaluate the imaging modalities. A larger study population could provide more comprehensive insights into the performance of these techniques in these specific patient groups.

## Conclusion

4DCT in conjunction with US and SeS allows more sensitive and accurate detection of parathyroid adenomas, compared to US and SeS alone. US should be used as a first-line strategy for identifying adenomas, as a positive result is typically reliable, and it does not involve radiation exposure. Nevertheless, a negative result should prompt careful consideration of the clinical context, and additional diagnostic steps—such as other imaging modalities or intra-operative exploration—may be warranted.

These findings should, however, be interpreted in the context of the study’s retrospective design and selected patient population. Prospective studies with larger cohorts are needed to validate the results and further assess the performance of these imaging modalities.

## Data Availability

The anonymized datasets used and/or analyzed during the current study are available from the corresponding author on reasonable request.

## Abbreviations

4DCT: Four-dimensional computed tomography
BNE: Bilateral neck exploration
CI: Confidence intervals
DLP: Dose-length product
MAD: Mean absolute deviation
MIP: Minimally invasive parathyroidectomy
NPV: Negative predictive value
PHPT: Primary hyperparathyroidism
PPV: Positive predictive value
PTH: Parathyroid hormone
SeS: Sestamibi scintigraphy
SPECT: Single-photon emission computed tomography
US: Ultrasonography
